# Loss of 11βHSD1 enhances glycolysis, facilitates intrahepatic metastasis, and indicates poor prognosis in hepatocellular carcinoma

**DOI:** 10.18632/oncotarget.6661

**Published:** 2015-12-18

**Authors:** Xu Liu, Xiao-long Tan, Meng Xia, Chao Wu, Jia Song, Jing-jing Wu, Arian Laurence, Qing-guo Xie, Ming-zhi Zhang, Hui-fang Liang, Bi-xiang Zhang, Xiao-ping Chen

**Affiliations:** ^1^ Hepatic Surgery Centre, Tongji Hospital, Tongji Medical College, Huazhong University of Science and Technology, Wuhan, Hubei, China; ^2^ Department of Hepatobiliary and Pancreatic Surgery, Peking University Shenzhen Hospital, Shenzhen, Guangdong, China; ^3^ The Newcastle upon Tyne Hospitals NHS Foundation Trust, Freeman Hospital, Newcastle upon Tyne, UK; ^4^ Department of Biomedical Engineering, and Wuhan National Laboratory for Optoelectronics (WNLO), Huazhong University of Science and Technology, Wuhan, Hubei, China; ^5^ Department of Cancer Biology, Vanderbilt-Ingram Cancer Center, Vanderbilt University School of Medicine, Nashville, TN, USA

**Keywords:** 11beta-hydroxysteroid dehydrogenase type 1, aerobic glycolysis, hepatocellular carcinoma, intrahepatic metastasis, prognostic biomarker

## Abstract

11Beta-hydroxysteroid dehydrogenase type 1 (11βHSD1), converting glucocorticoids from hormonally inactive cortisone to active cortisol, plays an essential role in glucose homeostasis. Accumulating evidence suggests that enhanced glycolytic activity is closely associated with postoperative recurrence and prognosis of hepatocellular carcinoma (HCC). Whether 11βHSD1 contributes to HCC metastasis and recurrence remains unclear. Here we found that expression of 11βHSD1 in human HCC (310 pairs) was frequently decreased compared to the adjacent non-neoplastic liver tissues (ANT), which correlated well with the intrahepatic-metastatic index, serum glycemia, and other malignant clinicopathological characteristics of HCC and predicted poor prognosis. Knockdown of 11βHSD1 in BEL-7402 cells drastically reduced the pH of culture medium and induced cell death. Meanwhile, overexpression of 11βHSD1 in SMMC-7721 HCC cells resulted in repression of cell migration, invasion, angiogenesis, and proliferation *in vitro*. When transferred into BALB/c nude mice, 11βHSD1 overexpression resulted in decreased intrahepatic metastasis, angiogenesis, and tumor size. F-18-2-fluoro-2-deoxyglucose accumulation assay measured by positron emission tomography elucidated that 11βHSD1 reduced glucose uptake and glycolysis in SMMC-7721 cells *in vitro*, and intrahepatic metastasis foci and subcutaneous tumor growth *in vivo*. We showed that 11βHSD1 repressed cell metastasis, angiogenesis and proliferation of HCC by causing disruption of glycolysis via the HIF-1α and c-MYC pathways. In conclusion, 11βHSD1 inhibits the intrahepatic metastasis of HCC via restriction of tumor glycolysis activity and may serve as a prognostic biomarker for patients.

## INTRODUCTION

Hepatocellular carcinoma (HCC) is one of the most prevalent malignant neoplasms and the third leading cause of cancer death worldwide [1]. Recurrence and metastasis predominantly contribute to the high mortality of HCC patients after curative resection [2]. However, little is known of the molecular mechanisms underlying recurrence or metastasis, a process that is an important contributor to the poor prognosis of HCC. Thus, deciphering these mechanisms is critical to designing novel therapies that will improve patient survival.

Aberrant glucose metabolism, aerobic glycolysis, or the “Warburg effect” is a hallmark of human cancers. Even in the presence of abundant oxygen, cancer cells largely rely on the glycolytic pathway rather than the tricarboxylic acid (TCA) cycle and mitochondrial oxidative phosphorylation [3, 4]. It is now understood that cancer cells have different metabolic demands than normal cells, and adjust their use of metabolites to meet those demands. Instead of a predominant program for effective production of adenosine triphosphate, proliferating tumor cells depend on a metabolic program of aerobic glycolysis to sustain anabolic production of biomass [5, 6]. Elevated glucose uptake and glycolysis is associated with poor prognosis of many tumors [6, 7], supporting the idea that metabolic adaptations might contribute to the malignant phenotype [8]. However, the molecular mechanisms leading to glycolysis enhancement in tumor cells are still not well elucidated.

Glucocorticoids (GCs) are a family of steroid hormones that are major regulators of glucose metabolism. They serve to increase and maintain normal concentrations of glucose in blood [9, 10]. GCs have been shown to repress solid tumor growth, reduce tumor mass, and prevent metastasis by blocking angiogenesis [11-13]. The action of GCs on target tissues is determined by nuclear-receptor density and intracellular metabolism by the two isozymes of 11beta-hydroxysteroid dehydrogenase (11βHSD), 11βHSD1 and 2, which catalyze interconversion of active cortisol (in humans) and corticosterone (in rodents) with inert cortisone and 11-dehydrocorticosterone [14].

11βHSD1 is predominantly but not exclusively an 11β-reductase in intact cells, regenerating and amplifying the action of GCs. 11βHSD2 is a dehydrogenase of high affinity that potently inactivates GCs [14, 15]. 11βHSD1 is principally expressed in the liver and plays a causal role in metabolic syndromes [16, 17]. In contrast, 11βHSD2 is largely restricted to the classical aldosterone (mineralocorticoid)-targeted tissues such as distal nephrons, and is crucial to blood pressure regulation [18, 19]. 11βHSDs have been reported to influence cell proliferation and neoplasia [20].

Notwithstanding the expression and activity of 11βHSD1 in various tissues, this isozyme is remarkable for its absence in tumors and tumor-derived cell lines [20, 21]. For example, normal pituitary tissue shows strong expression of 11βHSD1, especially in growth hormone- and prolactin-secreting cells [22]. In contrast, its expression in pituitary adenomas is greatly decreased [22, 23]. In a similar manner, 11βHSD1 is clearly defined and expressed in bone biopsies and primary cultures of osteoblastic cells [24], but is diminished in osteoblastic cell lines derived from osteosarcomas [25]. Other reports have also documented reduced expression of 11βHSD1 in squamous cell carcinomas of the head and neck, compared with adjacent non-cancerous mucosal tissues [26].

Overall, despite its abundance in the liver, the importance of hepatic 11βHSD1 in the pathophysiology of HCC remains unclear. Furthermore, there are no relevant studies in the literature addressing the effect of 11βHSD1 on HCC cells from the perspective of invasion and metastasis. The aims of this study were to examine the effect of 11βHSD1 and its ability to suppress glycolysis on intrahepatic metastasis and recurrence in HCC. We found that 11βHSD1 inhibited glucose uptake and glycolysis, which was associated with inhibition of tumor cell migration, invasion, angiogenesis and growth, resulting in reduction of tumor recurrence and intrahepatic metastasis.

## RESULTS

### Frequently decreased expression levels of 11βHSD1 in human HCC tissues

**Figure 1:**
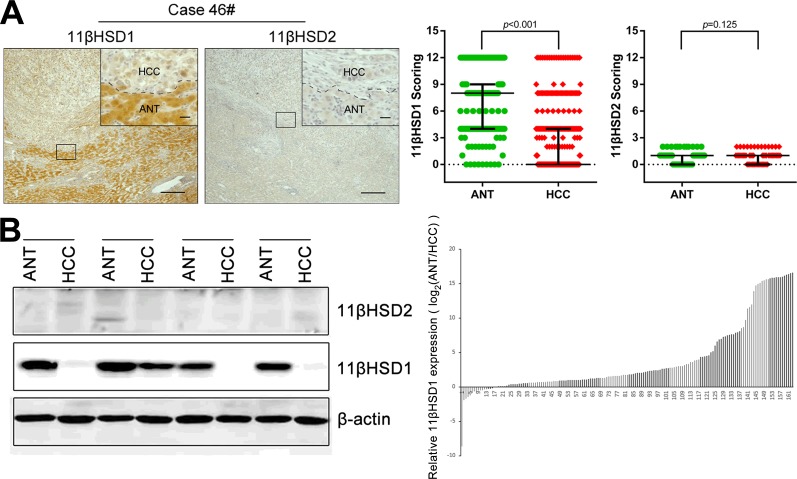
Loss of 11βHSD1 in hepatocellular carcinoma Immunohistochemical staining **A.** and western blotting analysis **B.** of 11βHSD1 and 11βHSD2 expression in paired HCT and ANT. Representative images are shown (**A.**, **B.**, left panel). Statistical analysis of 11βHSD1 and 11βHSD2 expression detected by IHC in HCC (**A.**, right panel) and 11βHSD1 expression detected by western blotting in HCC (**B.**, right panel). Scale bar, 50 μm (larger) or 20 μm (shorter).

To clarify the underlying role of 11βHSDs in HCC progression, we first examined the expression levels of 11βHSD1 and 2 protein in a cohort of 310 paired HCC tissues (HCT) and adjacent non-cancerous tissue (ANT) with clinicopathological features (Supporting Table S1). Immunohistochemistry (IHC) assays showed that the staining density of the 11βHSD1 protein in the ANT group was significantly more pronounced than that observed in the HCT group (*P*<0.001), while the difference in 11βHSD2 expression in ANT and HCC was not statistically significant (*P*=0.125). Representative IHC staining is shown in Figure 1A. Similar results were also confirmed in the western blotting assays containing 161 paired HCC and HCT samples. We found that 11βHSD1 expression was significantly reduced in the HCT compared with the matched ANT samples (Figure 1B, Supporting Figure S1A). These data all suggest that 11βHSD1, but not 11βHSD2, may play a protective role during HCC progression.

### Reduced 11βHSD1 expression in HCC predicts poor prognosis

**Figure 2:**
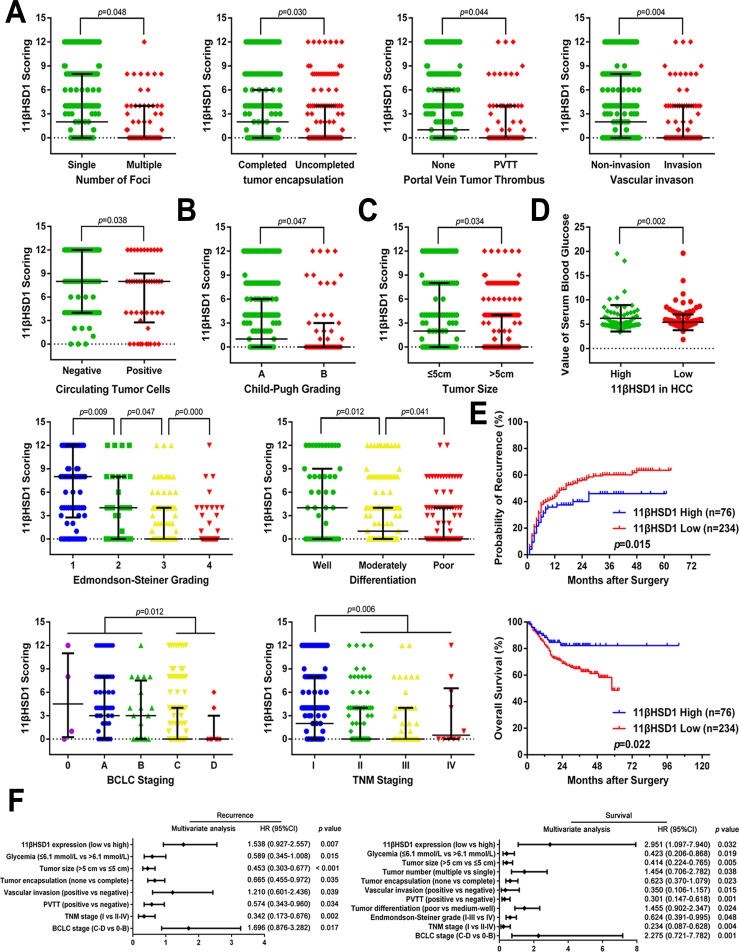
Reduced 11βHSD1 expression predicts aggressive clinicopathological characteristics and poor prognosis in HCC patients Relative expression scores of 11βHSD1 in 310 human HCC samples with or without multiple tumor nodes, encapsulation, PVTT, vascular invasion, CTCs **A**., advanced Child-Pugh grading, poor differentiation, advanced Edmondson-Steiner grading, TNM staging, BCLC staging **B**.; large tumor size **C**. and serum glycemia **D**. are shown as spot charts, with the middle bars representing the median, and the bottom and top of the bars representing the 25th and 75th percentiles, respectively; vertical bars represent the range of data. **E**. Kaplan-Meier analysis of the correlation between 11βHSD1 expression and the DFS or OS of HCC patients. **F**. A multivariate analysis of the hazard ratios (HRs) showed that the downregulation of 11βHSD1 in HCC may be an independent prognostic factor for the DFS and OS rates (by the Cox multivariate proportional hazard regression model). The HRs are presented as the means (95% confidence interval, 95% CI). The variables included in the multivariate analysis were selected using a univariate analysis.

We next sought to determine the clinical significance of 11βHSD1 expression in the development and progression of HCC and to determine whether 11βHSD1 expression in HCC is associated with disease recurrence and poor survival. The downregulation of 11βHSD1 in HCC was significantly correlated with several aggressive clinicopathological characteristics, including increased tumor number, vascular invasion, portal vein tumor thrombosis (PVTT), circulating tumor cells (CTCs) and lack of encapsulation (Figure 2A); advanced histological grade (Child-Pugh grades, Edmondson–Steiner grades, differentiation, BCLC stages, and TNM stages; Figure 2B), and tumor size (Figure 2C). More importantly, weak 11βHSD1 staining in HCC was directly associated with lower glycemic levels (Figure 2D, Supporting Figure S1B).

Based on these immunohistochemical results, all 310 patients with HCC were divided into two groups: high-expression (n=76) and low-expression groups (n=234). Patients in the low-expression group had both a shorter Disease-free survival (DFS; *P*=0.015) and a worse Overall survival (OS; *P*=0.022) compared with patients in the high-expression group (Figure 2E).

Multivariate analysis further indicated that 11βHSD1 reduction is one of the independent risk factors for prediction of patient prognosis (Figure 2F; Supporting Table S2). The group with lower 11βHSD1 expression also displayed a higher risk for cancer recurrence and a shorter OS time.

Taken together, 11βHSD1 expression levels inversely correlated with the malignant phenotype of HCC and the presence of metastasis while positively correlating with blood glucose concentration. These data indicate that the expression level of 11βHSD1 can be used as an independent factor for predicting HCC prognosis.

### Exogenous expression of 11βHSD1 significantly reduces HCC cell migration, invasion, and intrahepatic metastasis via decreased glycolysis

To explore the function of 11βHSD1 *in vitro*, we generated lentiviral constructs expressing 11βHSD1 (LV-HSD11B1) or control vectors to infect SMMC-7721 cells, (7721-HSD11B1 and 7721-vector, respectively) and siRNA against 11βHSD1 gene (*HSD11B1*) and scrambled siRNA were used to transiently knockdown *HSD11B1* in BEL-7402 cells (7402-siHSD11B1 and 7402-siSc, respectively) (Supporting Figure S2B). SMMC-7721 cells and BEL-7402 cells were chosen because these have the lowest or highest expression levels of endogenous 11βHSD1 compared with other HCC cell lines, respectively (Supporting Figure S2A). 7402-siHSD11B1 cells all died within 72 hours after transfection due to the extremely low pH of the culture medium (Supporting Figure S2C–S2D).

**Figure 3:**
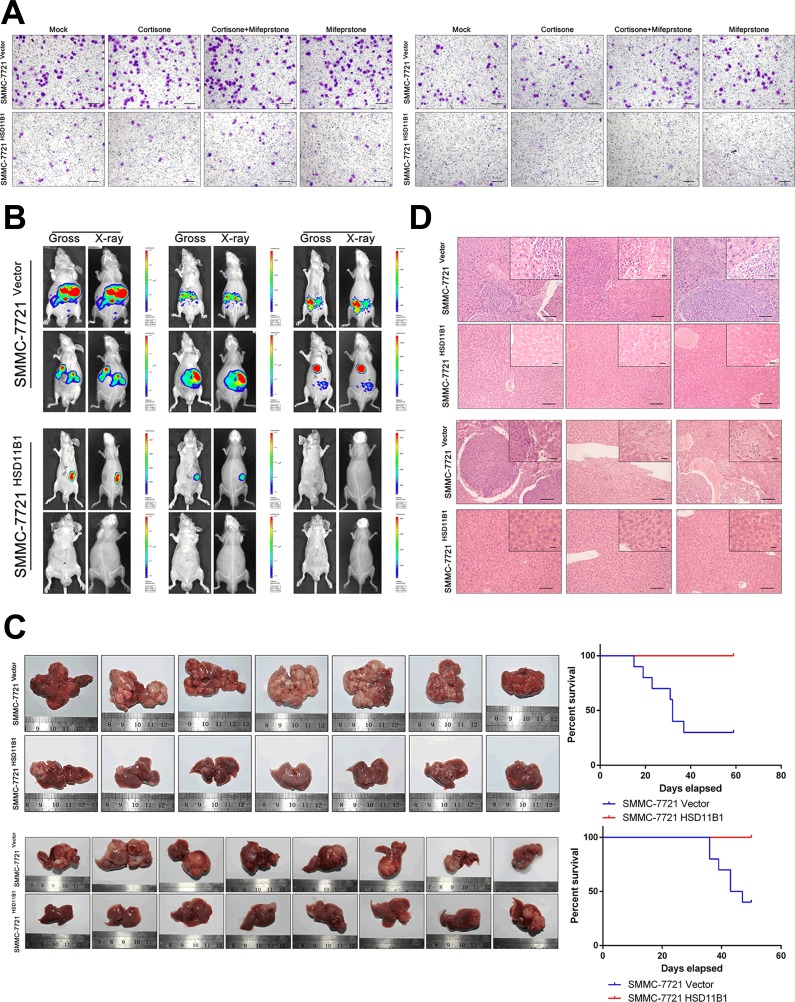
11βHSD1 reduces invasion and metastasis of HCC cells **A**. Overexpression of 11βHSD1 repressed SMMC-7721 cell migration (left panel) and invasion (right panel) in transwell assays. The results are representative of three independent experiments, *P* < 0.001. Scale bar, 50 μm. **B**. *In vivo* orthotopic tumor metastasis assay: luciferase-expressing cell lines (7721 luc-HSD11B1 and 7721 luc-vector) were transplanted into livers of nude mice. Tumor metastasis was monitored by bioluminescence imaging. Bioluminescent images showed the presence of expansive growth and intrahepatic metastases in mice implanted with indicated cells. **C**. Images of HCC tumors growing in the livers of nude mice administered a portal vein injection (upper, left panel) and orthotopic implantation (lower, left panel) of 7721-vector and 7721-HSD11B1 tumor masses. OS time of portal vein injection group (upper, right panel, *n* = 10; *P* < 0.01) and orthotopic implantation group (lower, right panel, *n* = 10; *P* < 0.001). **D**. Images showing representative hematoxylin and eosin staining of liver tissue samples from the portal vein injection (upper panel) and orthotopic implantation (lower panel) groups. Scale bar, 50 μm (larger) or 20 μm (shorter).

We then explored the effect of 11βHSD1 on the motility and invasiveness of SMMC-7721 cells. Transwell migration and Matrigel invasion assays revealed that exogenous 11βHSD1 expression significantly decreased cell mobility compared with the control HCC cells (Figure 3A).

To verify the capacity of 11βHSD1 to inhibit cell invasion *in vivo*, 7721-HSD11B1 and control cells were injected into the portal vein of nude mice. Eight weeks later, the mouse livers were stained with hematoxylin and eosin and liver micrometastases were microscopically evaluated. As shown in the upper panels of Figure 3C and 3D, 11βHSD1-expressing mice displayed fewer and smaller metastatic foci in the liver, and prolonged the survival time. The health condition of 11βHSD1-expressing mice was much better than that of control mice.

We further examined the role of 11βHSD1 in HCC metastasis by establishing an orthotopic tumor metastasis model in nude mice, which closely mimicked the process of human HCC metastasis after formation of a primary focus. SMMC-7721 cells stably expressing luciferase were established (7721-luc), and vector- and 11βHSD1-expressing 7721-luc subcutaneous tumors were transplanted into healthy animals. After 8 weeks, tumors of the control group mice had more intra-hepatic metastases (Figure 3B, Figure 3C lower panel), with more frequent invasive growth fronts with irregular tumor borders and tumor microsatellites, whereas those of the 11βHSD1-expressing mice more often had tumor growth fronts with more regular and less invasive borders (Figure 3D lower panel).

### Impaired glucose uptake and glycolysis confer the 11βHSD1-reduced metastasis capability of HCC cells

**Figure 4:**
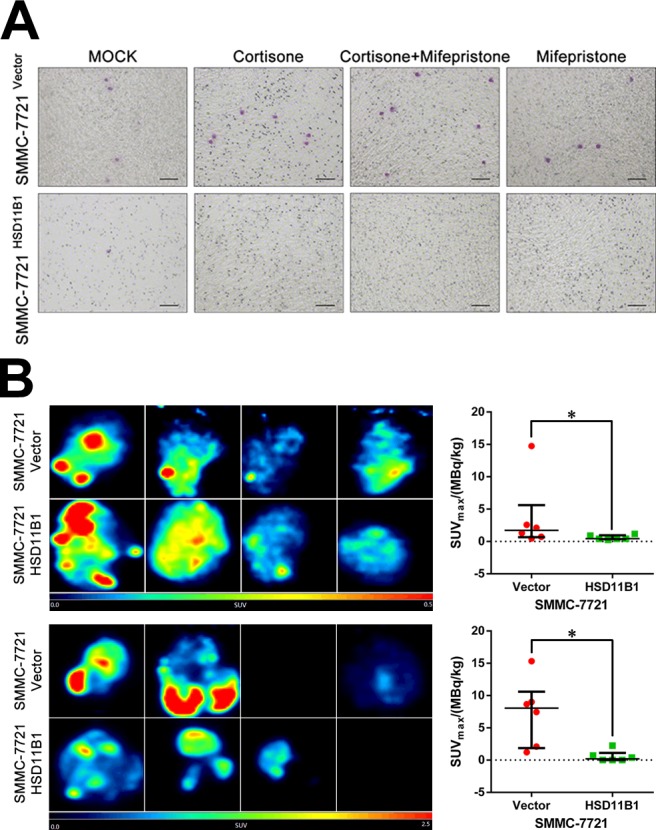
Impaired glycolysis confers 11βHSD1-repressed metastasis **A**. Elevated 11βHSD1 inhibited SMMC-7721 cell migration in 25 mM glucose-containing, serum-free culture medium. The results are representative of three independent experiments; scale bar, 50 μm. **B**. Representative images of ^18^F-FDG accumulation in the left lateral lobe of liver (upper panel) and resected metastasis foci (lower panel) of mice administered a portal vein injection. The SUV_max_ of ^18^F-FDG uptake in each group (*n* = 6) is presented as the mean ± SD, *P < 0.001.

Previous studies indicated that metastasis is under metabolic control [27, 28]. We hypothesized that 11βHSD1 suppresses invasion and metastasis of SMMC-7721 cells through regulation of glucose uptake and use. To investigate the effect of glucose metabolism on the motility of SMMC-7721 cells, we performed a transwell migration assay with a concentration gradient of glucose (0–50 mM) in serum-free culture media. With an increasing glucose concentration, there was a significant increase in the number of the SMMC-7721 cells that migrated through the transwell membrane (Supporting Figure S4A). This was reversed by the addition of the glycolysis inhibitor 2-deoxy-D-glucose (2DG; Supporting Figure S4B). We next compared 7721-vector with 7721-11βHSD1 and found that the presence of 11βHSD1 significantly inhibited migration through the transwell (Figure 4A).

Next, the left lateral lobes of the mouse livers were excised and assessed for tumor glucose uptake by positron emission tomography (PET). ^18^F-FDG uptake of 11βHSD1-expressing tumors was significantly decreased compared with the control tumors. When the metastatic foci were dissected, there was similar elevated ^18^F-FDG accumulation in tumors that lacked 11βHSD1 (Figure 4B).

### Enhanced expression of 11βHSD1 represses cancer-mediated angiogenesis

**Figure 5:**
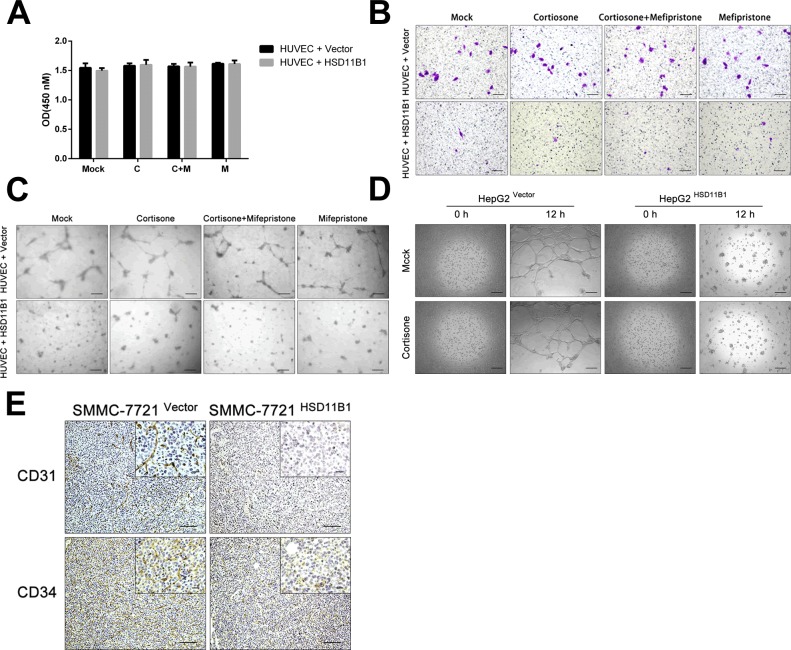
11βHSD1 attenuates hepatoma angiogenesis and vasculogenic mimicry **A**. HUVECs (1×10^3^ cells/well) were co-cultured on the bottom of a 0.4-μm transwell chamber with the indicated cell lines (7721-vector and 7721-HSD11B1). CCK-8 assay detected the proliferation of HUVECs. **B**. A migration assay was performed with HUVECs (1×10^4^ cells/well). HUVECs were co-cultured in the upper 8-μm inserts with indicated cells (7721-vector and 7721-HSD11B1) seeded onto the bottom chamber. Scale bar, 50 μm. **C**. The effect of 11βHSD1 on the capillary-like tube formation of HUVECs. HUVECs (2×10^4^ cells/wsell) were seeded onto Matrigel-coated 0.4-μm inserts (upper chamber) with indicated cells (7721-vector and 7721-HSD11B1) seeded onto the bottom. Representative images of HUVEC tube formation. Scale bar, 50 μm. **D**. Representative images of enhanced 11βHSD1 inhibited the vasculogenic mimicry potential of HepG2 cells (2×10^4^ cells/well). The results above are representative of three independent experiments. Scale bar, 50 μm. **E**. Immunohistochemical analysis of CD31 and CD34 protein levels in subcutaneous tumors formed by the indicated cell lines (7721-vector and 7721-HSD11B1), *n* = 6, scale bar, 50 μm (larger) or 20 μm (shorter).

Glycolysis is required to provide the necessary energy for endothelial cells to switch from a quiescent state to one capable of supporting angiogenesis [29], a process that is fundamental to tumor metastasis [30]. HUVECs were cultured in the top chamber and 7721-HSD11B1 or control SMMC-7721 cells were cultured in the lower chamber of a transwell plate. After 5 days, we found that there was no difference in the numbers of HUVEC cells (Figure 5A). When the position of the cells was reversed, the number of HUVEC cells that had migrated to the upper chamber was significantly increased if they were cultured with SMMC-7721 cells compared with 7721-HSD11B1 cells (Figure 5B). Furthermore, we found that HUVECs co-cultured with 7721-HSD11B1 cells formed significantly fewer angiogenic-sprouting tubes than those cultured with control SMMC-7721 cells (Figure 5C).

The HepG2 HCC cell line is able to spontaneously form vascular tubes when cultured in Matrigel. To investigate the effect of 11βHSD1 on this process, we compared wild-type HepG2 cells with HepG2-HSD11B1 overexpressing 11βHSD1 (Supporting Figure S2B). We found that HepG2-HSD11B1 cells had a significant reduction in the number of vascular tubes compared with wild-type HepG2 cells after 12 hours of culture in Matrigel (Figure 5D).

To determine the potential *in vivo* role of 11βHSD1 in tumor angiogenesis, we determined the expression of CD31 and CD34 by IHC in subcutaneous tumors derived from 7721-vector and 7721-HSD11B1 cells. CD31 and CD34 protein expression was lower in subcutaneous tumors derived from 7721-HSD11B1 as compared with those derived from control vector cells (Figure 5E).

### 11βHSD1 attenuates local growth potential of HCC cells both *in vitro* and *in vivo*

**Figure 6:**
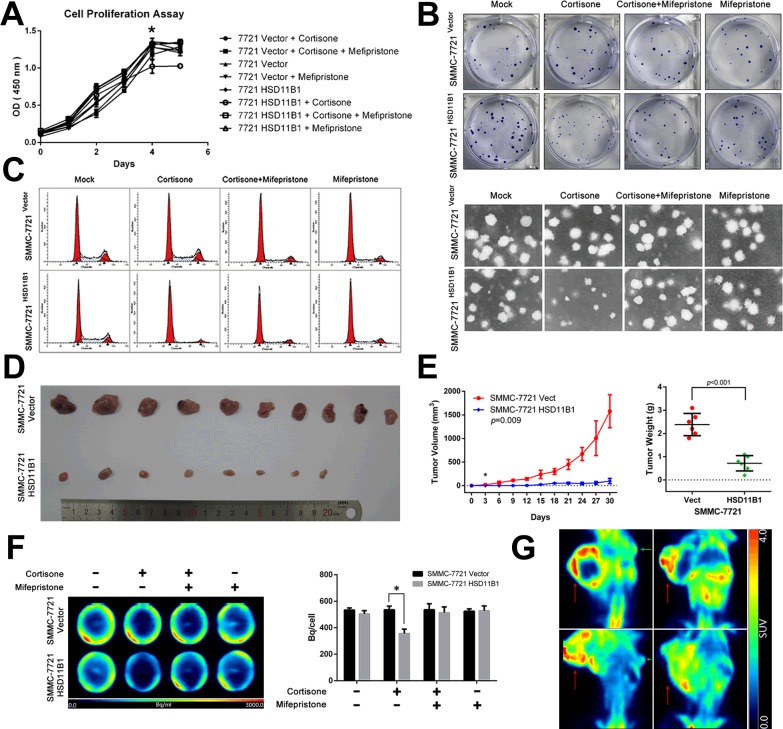
11βHSD1 represses proliferation and growth of HCC cells **A**. Following treatment with 10 μM cortisone, 11βHSD1 overexpression inhibited the proliferation of SMMC-7721 in a time-dependent manner, which could be attenuated by 1 μM mifepristone. **B**. Indicated cells were subjected to colony formation assays and soft agar colony formation assays. Representative images are shown. **C**. Cell cycle assay of indicated groups. The results are representative of three independent experiments. **D**. Images of subcutaneous tumors from 7721-vector and 7721-HSD11B1 groups are shown. **E**. Volume and weight of subcutaneous tumors from each group are shown. *n* = 10. **F**. Representative images of ^18^F-FDG accumulation in indicated groups *in vitro* (left panel). The results are representative of three independent experiments. Statistical analysis of ^18^F-FDG accumulation is shown (right panel), *P* < 0.001. **G**. Representative images of ^18^F-FDG accumulation in subcutaneous tumors from 7721-vector and 7721-HSD11B1 groups.

Local proliferation of tumor cells, an essential step in metastasis, is necessary for subsequent macrometastases and formation of a new tumor. Therefore, we examined the effect of 11βHSD1 on SMMC-7721 cell proliferation in the presence or absence of either cortisone, mifepristone, or both. After 5 days, the presence of cortisone resulted in a significant increase in the number of SMMC-7721 cells compared with 7721-HSD11B1 cells, which was reversed by mifepristone, an antagonist of the glucocorticoid receptor (Figure 6A, Supporting Figure S3A–S3C).

Next, we investigated the ability of cortisone to decrease colony formation. The experiment was repeated using just 100 cells preconditioned in media or 5000 cells preconditioned in soft agar: after 14 days, the number of individual cell colonies was counted. The presence of cortisone resulted in a significant increase in the number of SMMC-7721 colonies compared with 7721-HSD11B1 colonies, which was reversed by mifepristone (Figure 6B).

We next determined the mechanism by which 11βHSD1 inhibited cell-cycle progression. Wild-type SMMC-7721 cells or 11βHSD1-expressing 7721-11βHSD1 cells were stimulated with either cortisone, mifepristone, or both. Cells were lysed and the cell-cycle phase distribution was determined. We found that in the presence of cortisone, a greater proportion of 7721-HSD11B1 cells was arrested in G0/G1 compared with SMMC-7721 cells, which was reversed by mifepristone (Figure 6C).

To explore the effect of glucose on the proliferation of HCC cells, we cultured SMMC-7721 cells (1000/well) in the presence of increasing concentrations of glucose. After 5 days, the number of SMMC-7721 cells was significantly increased with increasing concentrations of glucose (Supporting Figure S4C), which was reversed in the presence of 2-DG (Supporting Figure S4D). We next investigated the effect of 11βHSD1 on SMMC-7721 glucose uptake in the presence or absence of either cortisone, mifepristone, or both. After 2 days, cells (5×10^5^/well) were treated with ^18^F-FDG, and its uptake was measured by PET. We found a significant increase in the absorption of ^18^F-FDG by SMMC-7721 cells compared with 7721-HSD11B1 cells in the presence of cortisone, which was reversed by mifepristone (Figure 6F).

To further evaluate the tumor-repressing properties of 11βHSD1 *in vivo*, 7721-HSD11B1 and control SMMC-7721 cells were subcutaneously inoculated into nude mice. Mice that received 7721-HSD11B1 cells exhibited a delay in the appearance of subcutaneous tumors, and a reduction in tumor size, tumor weight, and tumor 18F-FDG accumulation per milligram of tumor tissue, compared with mice that received SMMC-7721 cells (Figure 6D, 6E, and 6G).

### HIF1-α and c-MYC contribute to the repressing role of 11βHSD1 on proliferation and metastasis

**Figure 7:**
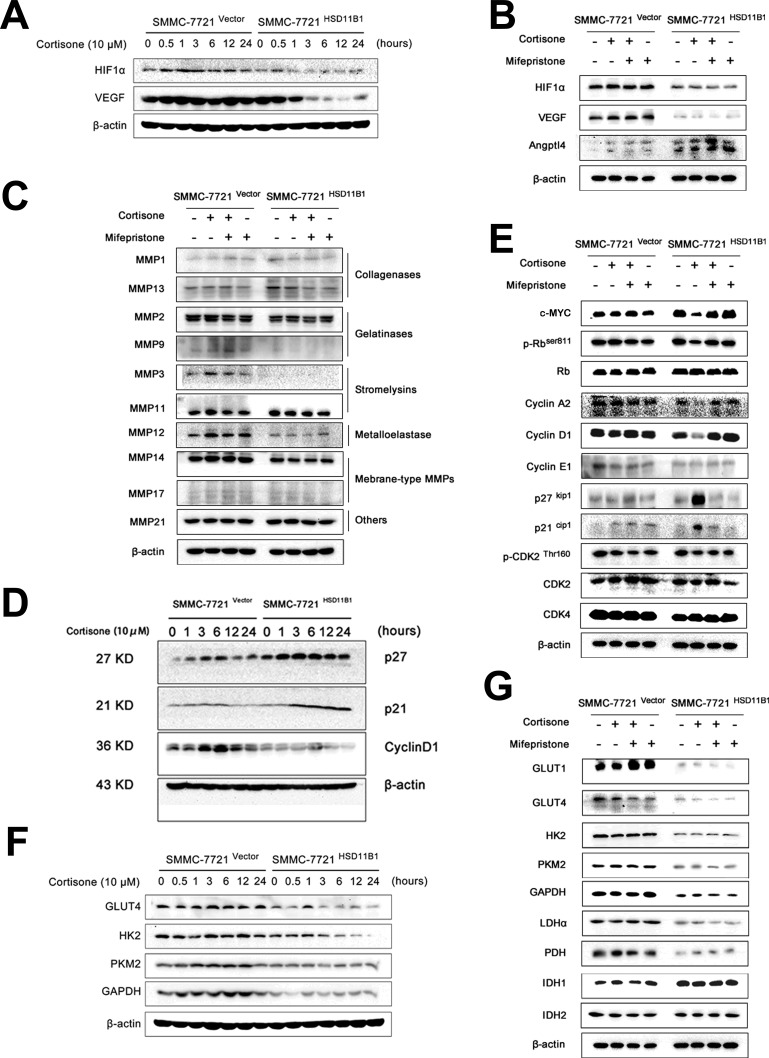
Glycolysis restriction is indispensable for 11βHSD1-mediated metastasis blocking Protein lysates from cells treated with 10 μM cortisone for indicated time points were subjected to western blotting. Representative images show expression of proteins related to metastasis **A**., the cell cycle **D**., and enzymes involved in glycolysis **F**. Protein lysates from cells treated with indicated chemicals (cortisone, 10 μM; mifepristone, 1 μM) for 6 h were subjected to western blotting. Representative images show expression of proteins related to metastasis and angiogenesis **B**., invasion **C**., the cell cycle **E**., and enzymes involved in glycolysis **G**. β-Actin was used as a loading control. The results are representative of three independent experiments.

We determined the mechanism by which 11βHSD1 inhibited cell migration and invasion. 7721-11βHSD1 and control vector cells were stimulated with either cortisone, mifepristone, or both for 6 hours. Cells were lysed and the expression of the regulators of cell metastasis were determined by western blotting. Protein expression of HIF1-α, VEGF, MMP2, MMP3, MMP9, MMP12, and MMP14 was reduced and ANGPTL4 was elevated (Figure 7A–7C, Supporting Figure S5A–S5C).

We next measured a number of regulators of cell cycle by western blotting: the protein expression of the cyclin-dependent kinase inhibitors p21^Cip1^ and p27^Kip1^ increased significantly after 6 hours, and this was sustained until 24 hours after cortisone stimulation in 7721-HSD11B1 cells. Conversely, cyclin D1 expression decreased significantly 6 hours after exposure to cortisone. Moreover, 7721-11βHSD1 and control vector cells were stimulated with either cortisone, mifepristone or both for 6 hours. Cells were lysed, and expression of the regulators of the cell cycle were determined by western blotting. c-MYC, phosphorylation of Rb (Ser811) and cyclin A2 was also inhibited by cortisone; these all could be attenuated by mifepristone administration. In contrast, cortisone stimulation was unable to downregulate the extent of cyclin E1, cyclin-dependent kinase 2 (CDK2), p-CDK (Thr160), and cyclin-dependent kinase 4 (CDK4) expression in 7721-HSD11B1 cells (Figure 7D–7E, Supporting Figure S6A–S6B).

Simultaneously, we also found that overexpression of 11βHSD1 in SMMC-7721 cells led to reduced expression of critical regulators of glycolysis, GLUT1, GLUT4, HK2, GAPDH, PKM2, LDHα, and PDH, while IDH1 was increased (Figure 7F–7G, Supporting Figure S7A–S7B).

## DISCUSSION

The dismal prognosis of HCC has been attributed to postsurgical recurrence and metastasis-related development of *de novo* tumors [31, 32]. Hence, it is extraordinarily vital to explore the molecular mechanisms governing the pathogenesis of cancer metastasis in HCC. In this study, we first determined the significance and underlying mechanism for 11βHSD1 downregulation in HCC progression and metastasis. Repressed glycolysis is involved in 11βHSD1-restricted metastasis.

Analyzing the association of 11βHSD1 expression with pathological characteristics in 310 patients with HCC by IHC staining revealed a downregulation of 11βHSD1 expression in HCC compared with the paired ANT samples. In line with these data, public databases, such as Oncomine [33] and Oncogenomic Database [34], showed a significant decrease of 11βHSD1 mRNA expression in HCC [35, 36]. We further clarified a significant correlation of 11βHSD1 expression with tumor size, tumor number, capsular invasion, vascular invasion, PVTT, CTCs, and histological grade, which are all hallmarks for poor prognosis of HCC [37]. Kaplan–Meier analysis showed that patients with HCC who had low 11βHSD1 expression in general had worse prognosis than those with high expression. Furthermore, multivariate analysis revealed that the expression level of 11βHSD1 was an independent and significant predictor for prognosis and survival after curative resection. These all strongly suggested that 11βHSD1 is an attractive candidate for risk prognostication and therapy of HCC.

11βHSD1 is expressed in a number of tissues important for metabolic regulation, such as the liver, central nervous system, and adipose tissue [17]. 11βHSD1 regenerates cortisol, amplifying the intracellular glucocorticoid signal in target tissues. Abnormal expression of this isozyme contributes to the pathogenesis of many metabolic disorders [16, 38]. Here, we explored that downregulation of 11βHSD1 was associated with a decreased concentration of serum glucose in HCC patients. Recent research indicates that metabolic profiling analysis of liver tissues provides a holistic view of the metabolic features of HCC.[39] The rapid consumption of energy by the tumor cells upregulates glycolysis, represses the TCA cycle, and decreases concentrations of glucose, accordant with the “Warburg effect” [39-42]. Bo Wang et al., using a mouse model, fed a choline-deficient and amino acid-defined diet to induce liver tumors in the absence of any exogenous chemicals or virus, and found the serum glucose concentration of tumor-bearing mice was markedly decreased [43]. Furthermore, the global 11βHSD1-knockout mouse (GHKO) and liver-specific 11βHSD1-knockout mouse (LHKO) studies underlined the essentiality of hepatic 11βHSD1 in regulating hepatic glucose output. A high-fat diet led to GHKO and LHKO that had significantly lower fasting plasma glucose concentrations than weight-matched litter mates. GHKO and LHKO are able to resist the hyperglycemia observed in obese wild-type mice [44, 45]. Therefore, we hypothesize that the deprivation of 11βHSD1 in malignant hepatocytes implies that during the development and metastasis, HCC cells engage 11βHSD1 to remodel or reconstruct new metabolic networks.

The effect of 11βHSD1 on tumor invasion and metastasis was directly elucidated in our *in vitro* and *in vivo* studies. In both portal vein-injected and orthotopic xenografts, overexpression of 11βHSD1 generated fewer intrahepatic metastatic foci and lower levels of vascularization and angiogenesis, indicating their less aggressive and metastatic properties. To our knowledge, this is the first report that 11βHSD1 expression is critical for HCC metastasis, in addition to tumor proliferation and growth.

Our study reveals that HCC migration was enhanced with increasing glucose concentrations, suggesting that glucose plays an essential role both in HCC cell chemo-attraction and metastasis. We found that 11βHSD1 impaired the glycolysis-induced migratory potential of SMMC-7721 cells *in vitro* and repressed ^18^F-FDG accumulation in the metastatic foci *in vivo*. Meanwhile, 11βHSD1 knockdown in BEL-7402 results in acidosis-induced cell death. Furthermore, we identified that 11βHSD1 reduced the expression of many enzymes involved in metastasis (HIF-1α, VEGF, MMP2, MMP3, MMP9, MMP12, and MMP14), glucose uptake (GLUT1 and GLUT4) and glycolysis (HK2, GAPDH, PKM2, LDHα, and PDH), and increased expression of enzymes involved in TCA (IDH1) and that of a metastasis and glucose metabolism regulator (ANGPTL4). These all suggest that the ability of 11βHSD1 to prevent metastasis is dependent on its effect on glycolysis.

Tumors cannot grow beyond 2 mm^3^ in the absence of angiogenesis because of the limited diffusion of glucose, oxygen, and other nutrients [8, 46]. Tumor angiogenesis and neovascularization is one of the strategies by which tumors can overcome this stress, and is crucial for tumor expansion, invasion, and metastasis [47, 48]. Our results showed that overexpression 11βHSD1 in SMMC-7721 cells was able to repress the recruitment and tube formation of HUVECs *in vitro*, block angiogenesis and vascularization *in vivo*, reduce protein levels of HIF1-α and VEGF, and enhance the expression of ANGPTL4. Additionally, 11βHSD1 inhibited the vasculogenic mimicry (VM) potential of HepG2 cells. The unique structure of VM channels facilitates the hematogenous metastasis of tumor cells [49], and VM is correlated with poor clinical prognosis in patients [50]. Our findings demonstrated the ability of 11βHSD1 to inhibit angiogenesis, a form of metabolic adaptation and an advantage for metastasis.

In addition, we discovered that 11βHSD1 restrained the proliferation of HCC cells, which is dependent on the existence of cortisone, and could be attenuated by the GR inhibitor mifepristone. In subcutaneous xenografts, overexpression of 11βHSD1 was associated with significantly delayed tumor formation, reduction in tumor size, and decreased ^18^F-FDG uptake. 11βHSD1 upregulated the expression of cyclin-dependent kinase inhibitors (p21^Cip1^ and p27^Kip1^), and decreased the expression of proteins involved in the cell cycle (c-MYC, cyclin D1, cyclin A2, and p-Rb^Ser811^), which could be attenuated by mifepristone administration. Tumor cells are programmed to depend on aerobic glycolysis to support their proliferation and growth, which generates adenosine triphosphate and diverts glucose-derived carbon into macromolecular precursors for the synthesis of nucleotides, proteins, and lipids. This in turn facilitates the synthesis of macromolecules and organelles indispensable for assembly of new cells [5, 6].

HIF-1α and c-MYC are both crucial transcription factors that participate in the glycolytic switch, for example, modulating the expression of HK2, PKM2, LDHα, and PDK1, and in the induction of cell proliferation and metastasis [7, 51, 52]. It is demonstrated that c-MYC transactivates virtually all glycolytic enzyme genes, many of which are also activated by HIF-1α in hypoxia [51, 52]. For instance, c-MYC and HIF-1α could cooperatively activate HK2 and PDK1, which are important regulators of glycolytic metabolism and mitochondrial function [53]. Scanning chromatin immunoprecipitation (ChIP) assays for DNA binding of c-MYC and HIF-1α in HK2 and PDK1 genes demonstrated that c-MYC and HIF-1α independently bind different genomic regions.[51] These observations suggest that in the context of the Warburg effect, HIF-1α no longer inhibits c-MYC activity but instead the two cooperate to transactivate certain common target genes, such as *HK2* and *PDK1* [53].

Cytotoxic cancer treatment is based on drugs that unselectively kill proliferating cells and are therefore accompanied by many undesirable side effects. Drug targets that can repress invasion but leave cellular proliferation relatively spared may be capable of avoiding these side effects. Such targets may have the extra advantage of diminishing the selection for more resistant clones that occurs because of the elimination of treatment-sensitive cells [54]. Understanding the metabolic factors that are deeply connected with cell invasion may lead to novel anti-metastatic therapeutic opportunities.

In conclusion, we have identified 11βHSD1 as an important regulator that controls glucose uptake and glycolysis for HCC development and metastasis. In particular, the data have led us to propose that 11βHSD1 is a novel marker in the prognosis of HCC and a potential therapeutic target. Because 11βHSD1 is also downregulated in other types of cancers, including lung, breast, pituitary adenomas, and osteosarcomas, we believe that this tumor suppressor may be widely involved in approaches for cancer therapy.

## MATERIALS AND METHODS

### Patients, specimens, and follow-up

A total of 310 HCC tissue samples were collected from patients who underwent surgical resection at the Hepatic Surgery Centre, Tongji Hospital of Huazhong University of Science and Technology (HUST), Wuhan, China, from 2004 to 2014 (Supporting Table S1). The preoperative clinical diagnosis of HCC met the diagnostic criteria of the American Association for the Study of Liver Diseases.[31] All patients were followed up until October 2014, with a median observation time of 21 months. OS was defined as the interval between the dates of surgery and death. DFS was defined as the interval between the dates of surgery and recurrence; if recurrence was not diagnosed, patients were censored on the date of death or the last follow-up. The procedure of human sample collection was approved by the Ethic Committee of Tongji Hospital, HUST, and the study was conducted according to the Declaration of Helsinki Principles. Written informed consent was obtained from each patient.

### Animal studies

Four- to six-week-old male BALB/c (nu/nu) mice were housed under specific pathogen-free (SPF) conditions and cared for according to the institutional guidelines for animal care. All of the animal studies met the National Institutes of Health guidelines (NIH publication 86–23 revised 1985) and were approved by the Committee on the Ethics of Animal Experiments of Tongji Medical College, HUST.

For *in vivo* tumorigenicity assays, 1×10^6^ 11βHSD1-expressing SMMC-7721 cells and control vector cells were injected subcutaneously into the right flanks (7721-HSD11B1) and left flanks (7721-vector) of nude mice. Animals were inspected every 3 days for tumor development. Growing tumors were measured using Vernier calipers, and tumor volume was calculated using the formula length×width^2^×0.5, which approximates the volume of an elliptical solid.

For the *in situ* tumor-transplanted metastasis model, tumor cells were first injected subcutaneously into the flank region of 6-week-old male BALB/c nude mice. When the subcutaneous tumor reached approximately 1 cm in length, it was removed, minced into small pieces of equal volume (1 mm^3^), and transplanted into the livers of nude mice (16 mice per group). Three weeks post transplantation, the mice were subjected to *in vivo* luciferase analysis every week to monitor metastasis. Six mice of each group were sacrificed 6 weeks later. Their liver tissues were dissected, fixed, and prepared for histological examination. The remaining mice were observed for survival analysis with 8 weeks (60 days) as the cut-off.

For the portal vein injected metastasis model, 6-week-old male BALB/c nude mice were randomized into two groups (16 mice per group) and inoculated with tumor cells (2×10^6^) into the portal vein. Six mice of each group were sacrificed 8 weeks after inoculation, and metastatic tumor colonies in the liver were measured. The remaining mice were observed for survival analysis with 8 weeks (50 days) as the cut-off.

### FDG-PET analysis

Tumor-bearing mice were injected with F-18-2-fluoro-2-deoxyglucose (^18^F-FDG) intraperitoneally after 24 hours of fasting. After 40 min of ^18^F-FDG uptake, mice were anesthetized with chloral hydrate and imaged for 15 min by a prototype-dedicated small-animal positron emission tomography (PET; Trans-PET BioCaliburn LH system) scanner developed at the Xie Laboratory, HUST. For quantitative analysis, irregular regions of interest were placed over the most intense area of ^18^F-FDG accumulation. The maximum standardized uptake value (SUV_max_) was calculated using the following formula: maximum pixel value with the decay-corrected region-of-interest activity (MBq/kg)/(injected dose [MBq]/weight [kg]). The PET images were evaluated by two experienced nuclear medicine physicians.

### Tube formation assay and vasculogenic mimicry assay

For the tube formation assay, growth factor-reduced Matrigel (BD Biosciences, NJ, USA) was placed in the 0.4-μm insert (upper chamber) of a transwell (Corning Costar, NY, USA) and allowed to form a gel at 37°C for 30 min. HUVECs (2×10^4^ cells) were added into the upper chamber while HCC cells were added to the lower chamber, and incubated in serum-free culture medium at 37°C for 24 hours. Endothelial tubes were examined under a light microscope every 4 hours by inspecting the branch points.

For the vasculogenic mimicry assay, HCC cells (2×10^4^ cells/well) were seeded onto the Matrigel-coated bottom of a 96-well plate (Corning Costar, NY, USA) and incubated at 37°C for 12 hours. Tumor cell-formed tubes were examined under a light microscope every 4 hours by inspecting the branch points.

### Statistical analyses

Statistical analyses were performed using SPSS 13.0 (SPSS Inc., Chicago, IL, USA) or Prism 5.0 (GraphPad Software, La Jolla, CA, USA) software. Quantitative data were performed by a two-tailed Student t-test, analysis of variance with Bonferroni post-hoc test, or a nonparametric test such as the Wilcoxon signed-rank test, Mann–Whitney U test or Spearman rank correlation test. Kaplan–Meier and log-rank analyses were used to assess the survival between subgroups. A Cox proportional hazards model was used to determine the independent factors of survival and recurrence based on the variables selected in univariate and multivariate analyses. A value of *P*<0.05 was considered statistically significant.

A detailed description of additional materials and methods can be found in the Supporting Information.

## SUPPLEMENTARY MATERIAL METHODS, FIGURES AND TABLES


