# Supplementary material for: Loss of 11βHSD1 enhances glycolysis, facilitates intrahepatic metastasis, and indicates poor prognosis in hepatocellular carcinoma
Source: Oncotarget. 2015 Dec 18;7(2):2038–53. doi: 10.18632/oncotarget.6661 (PMC4811515; doi:10.18632/oncotarget.6661)
Supplement: Supplementary file 1 [file oncotarget-07-2038-s001.pdf]

Loss of 11βHSD1 enhances glycolysis, facilitates intrahepatic metastasis, and indicates poor prognosis in hepatocellular carcinoma

Supplementary Material

Methods

Immunohistochemistry

Sections were deparaffinized by standard process. Sections underwent microwave heat antigen retrieval in 10mM Tris Base, 1mM EDTA Solution, pH 9.0. Endogenous peroxidase activity was blocked with 3% H_2O_2 in methanol. Then sections were incubated with indicated antibody at 4°C overnight, washed with PBS and subsequently incubated with EnVision kit (DAKO Denmark) at room temperature. Peroxidase activity was detected using 3’3’diaminobenzidine tetrahydrochloride (DAB, DAKO Denmark) and counterstained with haematoxylin (Sigma-Aldrich, MO, USA).

Cell lines and Cell Culture

HCC cell lines with different metastatic potential (MHCC97L, MHCC97H, and HCCLM3) were obtained from Liver Cancer Institute, Zhongshan Hospital, Fudan University, Shanghai, China. Human HCC cell line HLE and HLF were kindly provided by Shanshan Wang and Gang Li (Department of Molecular Biology, Peking University Health Science Center, Beijing, China). Human fetal liver cell line HL-7702, hepatoma cell line HepG2, HCC cell lines Hep3B, Huh7, SK-Hep1, SMMC-7721 and BEL-7402 were purchased from China Center for Type Culture Collection (CCTCC, Wuhan, China). The cell line from noncancerous liver tissue QSG-7701 and HCC cell line PLC/PRF-5 were purchased from cell bank of Chinese Academy of Sciences (Shanghai, China). All cell lines were maintained in DMEM (Gibco, Thermo Fisher Scientific Inc., MA, USA) medium supplemented with 10% FBS (Gibco, Thermo Fisher Scientific Inc., MA, USA) and were incubated at least 12 hours in 0.2% serum for serum starvation before their

use in experiments.

Transient RNA interference

Small-interfering RNA (siRNA) duplexes targeting homo sapiens hydroxysteroid (11-beta) dehydrogenase 1 (HSD11B1, NM_005525.3) and scrambled siRNA were designed and synthesized by Life Technologies (siHSD11B1, s6932#, Silencer® siRNA, Life Technologies, Carlsbad, CA). Transfection of the siRNA into cells was performed using Lipofectamine LTX and Plus (Life Technologies, Carlsbad, CA) according to the manufacturer's instructions. The transfected cells were termed as 7402-siHSD11B1 or 7402-siSc, respectively.

Western blotting assay

Protein extracted was separated by SDS-PAGE and transferred to polyvinylidene fluoride (PVDF) membrane (F. Hoffmann-La Roche Ltd, Basel, CH). The membrane was probed with specific primary antibody, followed by incubation with a horseradish peroxidase (HRP)-conjugated anti-mouse or anti-rabbit secondary antibody (Jackson ImmunoResearch, USA). Detection was performed using ChemiDoc™ Imaging Systems (Bio-Rad Laboratories Co., Ltd., California, USA).

In Vitro Cell Behavior Assay

For cell proliferation assays, indicated cells were cultured in 96-well plates for various time periods. Cell Counting was measured using WST-8 (Dojindo Laboratories, Kumamoto, Japan) with a plate reader (Bio-Tek Elx 800, USA).

For colony formation assays, cells (100 cells/well) were plated in each well of a 6-well plate for 14 days, and the numbers of colonies greater than 100 μm in diameter were counted and analyzed by ChemiDoc™ Imaging Systems (Bio-Rad Laboratories Co., Ltd., California, USA).

For soft agarose assay, briefly, cells (0.5×10^4) were suspended in 1ml of 0.4% agarose

containing 10% FBS and then plated on top of 1ml of semi-solid 0.8% agarose in 6-well plate. Cells were treated with cortisone (10 μM), Mifepristone (1 mM) or both every 48 hours for 2 weeks. Colonies grown on soft agarose were counted and pictures of colonies were shown.

For transwell cell migration and invasion assays, tumor cells (2×10^4) in 0.2 ml serum-free medium were seeded in the 8μm insert (upper chamber) of a transwell (Corning Costar, NY, USA). The lower chamber was added with 0.5 ml medium containing 10% FBS. After 24 hours, cells remaining in the upper part of the transwell were removed with a cotton swab. Migrated cells were then stained with 0.5% Crystal Violet and the number of cells was counted per high field (×200) with a Nikon microscope. Transwell chamber inserts were pre-coated with Matrigel (BD Biosciences, NJ, USA) if cell invasion assays were performed. After 48 hours invaded cells were counted as invasion assay.

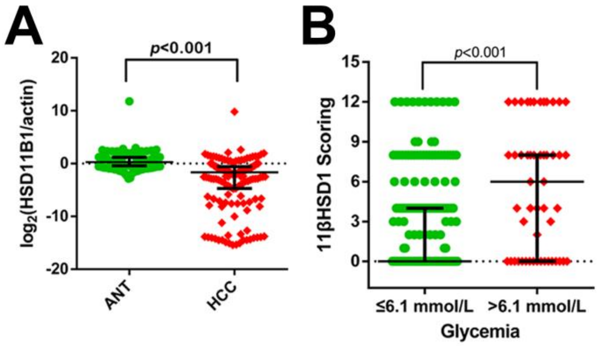

Supporting Figure S1: Reduced expression of 11βHSD1 in HCC is associated with a decreased serum glycemia. (A) 11βHSD1 expression in 161 pairs of HCT and ANT were detected by western blotting. 11βHSD1 bands of HCT and ANT were quantified and are shown in the spot chart after being normalized to their own actin. (B) Relative expression scores of 11βHSD1 in 310 human HCT with or without serum blood glucose are shown as spot charts, with the middle bars representing the median; the bottom and top of the bars represent the 25th and 75th percentiles, respectively; vertical bars represent the range of data.

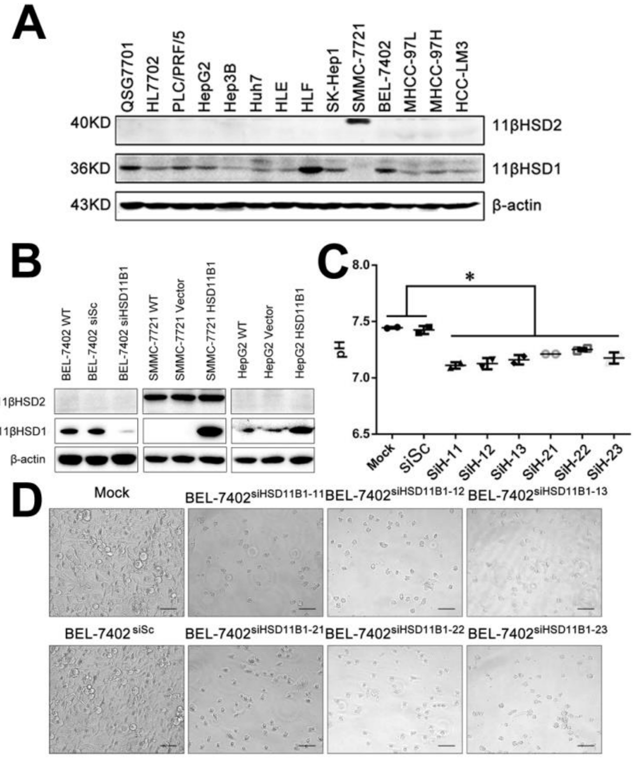

Supporting Figure S2: 11βHSD1 expression pattern in liver and hepatoma cell lines and knockdown 11βHSD1 in BEL-7402 cells induces acidosis and cell death. (A) Western blotting analysis of 11βHSD1 expression in different liver and hepatoma cell lines (n=3). Representative image is shown. (B) Increased 11βHSD1 protein expression in SMMC-7721 and HepG2 cells 48 hours after transfection with lentivirus containing the entire open reading frame of human 11βHSD1 cDNA. Reduced 11βHSD1 protein expression in BEL-7402 48 hours after transient transfection with HSD11B1-siRNA. (C) pH value of wild type BEL-7402, 7402-siSc, and each group of 7402-siHSD11B1 72 hours after transient transfection with HSD11B1-siRNA, *P<0.05. (D) Representative image of wild type BEL-7402, 7402- siSc, and each group of 7402-

siHSD11B1 72 hours after transient transfection with HSD11B1-siRNA. Scale bar, 50 μm.

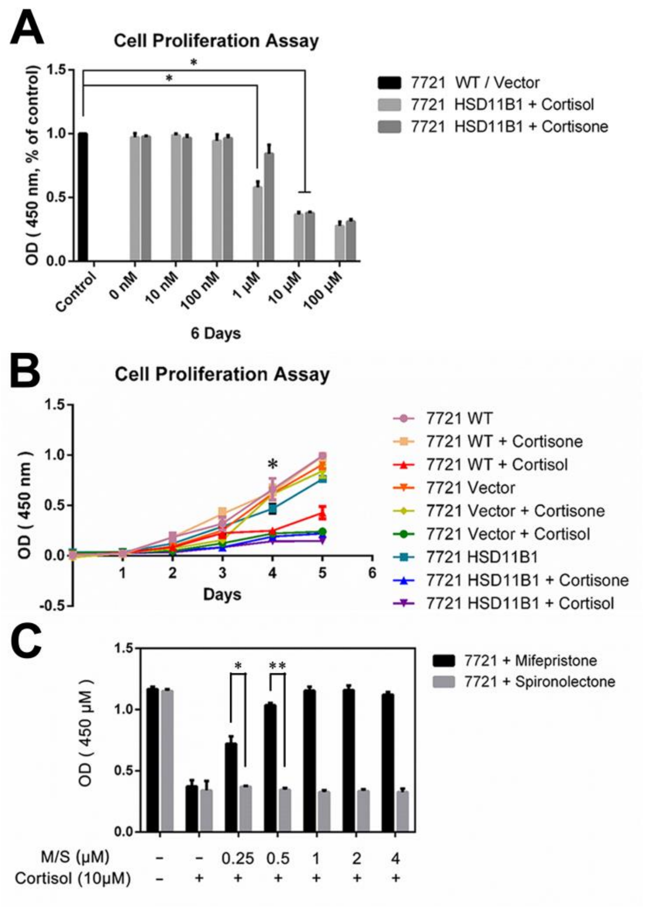

Supporting Figure S3: 11βHSD1 inhibits proliferation potential of SMMC-7721 in the presence of cortisone. (A) Treated with cortisone for 6 days, overexpression of 11βHSD1 inhibited the proliferation of SMMC-7721 in dose-dependent manner. Optical density (OD) of each group is shown with mean ± standard deviation of the mean of three independent experiments, **P*<0.01, ***P*<0.001. (B) Treated with 10 μM cortisone, overexpression of 11βHSD1 inhibited the proliferation of SMMC-7721 in a time-dependent manner. OD of each group is shown with mean ± standard deviation of the mean of three independent experiments, **P*<0.001. (C) Mifepristone attenuated the cytostasis of 11βHSD1 in a dose-dependent manner, treated with 10 μM cortisone, **P*<0.01, ***P*<0.001.

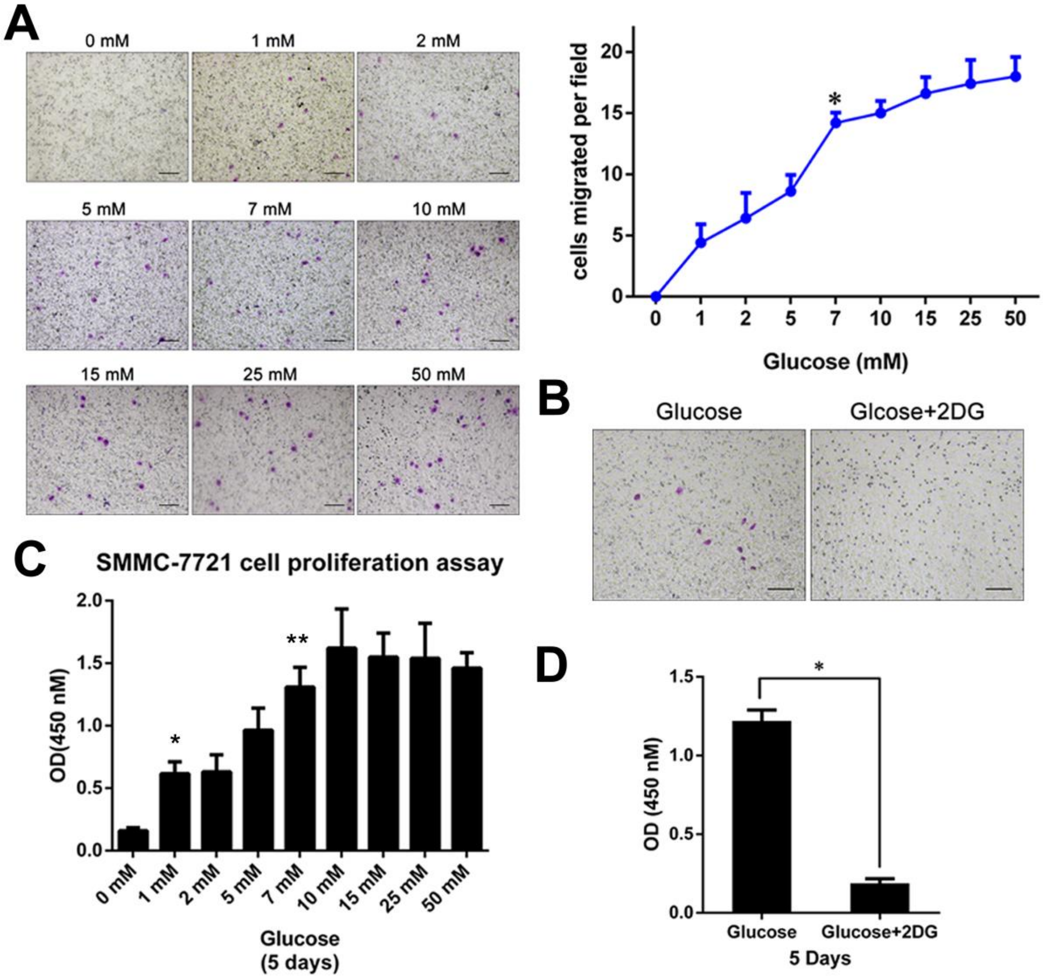

Supporting Figure S4: Glycolysis confers the motility and proliferation capability of SMMC-7721. (A) Cell transwell migration assay of SMMC-7721 cells performed in serum-free culture medium with increasing concentrations of glucose, *P<0.01, Scale bar, 50 μm. (B) Cell transwell migration assay of SMMC-7721 cells performed in serum-free culture medium with 10 mM glucose with or without 2DG (10mM) treatment, Scale bar, 50 μm. (C) CCK-8 assay of SMMC-7721 cells performed with increasing concentrations of glucose for 5 days. OD of each

group is shown with mean ± standard deviation of the mean of three independent experiments, **P*<0.01, ***P*<0.001. (D) CCK-8 assay of SMMC-7721 cells performed with 10mM glucose with or without 2DG (10mM) treatment. OD of each group is shown with mean ± standard deviation of the mean of three independent experiments, **P*<0.01.

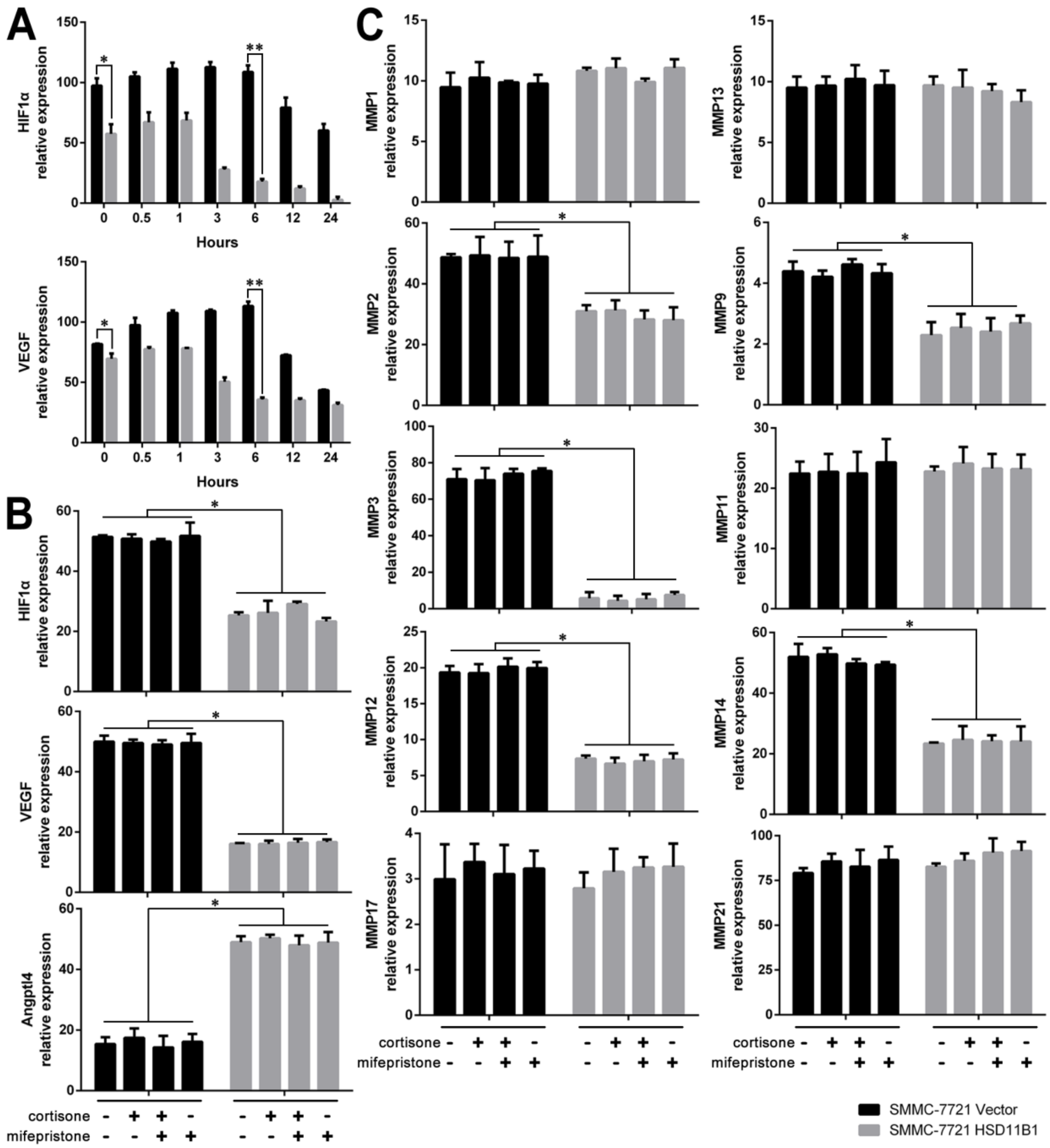

Supporting Figure S5: 11βHSD1 inhibits the expression of the regulators of cell metastasis in SMMC-7721 cells, related to Figure 7. (A) Statistical analysis of western blotting as described in Figure. 7A. (B) Statistical analysis of western blotting as described in Figure. 7B. (C) Statistical analysis of western blotting as described in Figure. 7C. Relative OD of each group is shown with mean ± standard deviation of the mean of three independent experiments, **P*<0.01, ***P*<0.001.

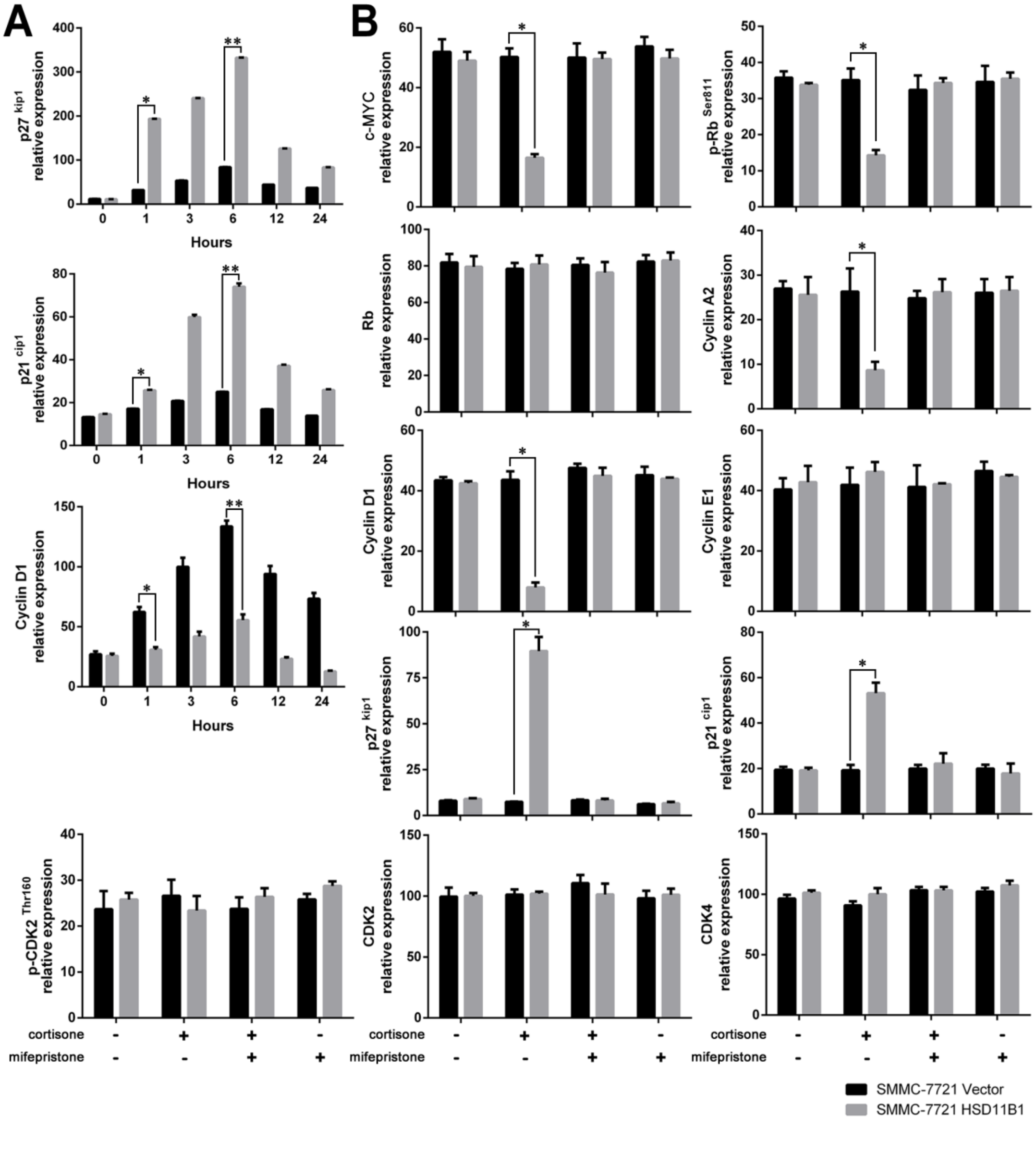
A
B
p27 kip1 relative expression
p21 cip1 relative expression
Cyclin D1 relative expression
Hours
0 1 3 6 12 24
c-MYC relative expression
p-Rb Ser811 relative expression
Rb relative expression
Cyclin A2 relative expression
Cyclin D1 relative expression
Cyclin E1 relative expression
p27 kip1 relative expression
p21 cip1 relative expression
p-CDK2 Thr160 relative expression
CDK2 relative expression
CDK4 relative expression
cortisone
mifepristone
SMMC-7721 Vector
SMMC-7721 HSD11B1

Supporting Figure S6: 11βHSD1 inhibits the expression of the regulators of cell cycle in SMMC-7721 cells, related to Figure 7. (A) Statistical analysis of western blotting as described in Figure. 7D. (B) Statistical analysis of western blotting as described in Figure. 7E. Relative OD of each group is shown with mean ± standard deviation of the mean of three independent experiments, **P*<0.01, ***P*<0.001.

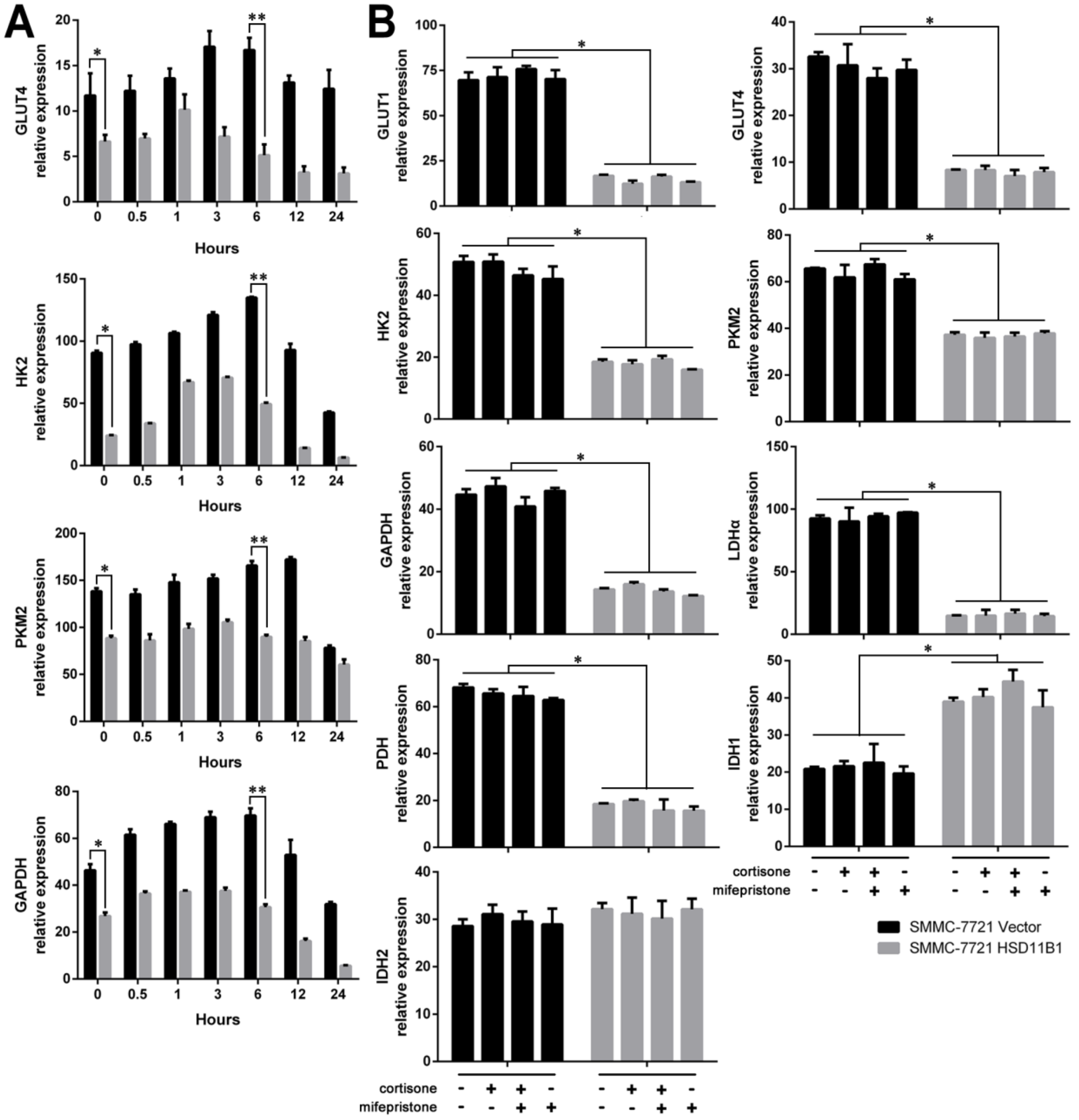

Supporting Figure S7: 11βHSD1 inhibits the expression of critical regulators of glycolysis in SMMC-7721 cells, related to Figure 7. (A) Statistical analysis of western blotting as described in Figure. 7F. (B) Statistical analysis of western blotting as described in Figure. 7G. Relative OD of each group is shown with mean ± standard deviation of the mean of three independent experiments, *P<0.01, **P<0.001.

Supplementary Table 1. Correlation between the Factors and Clinicopathologic Characteristics in HCC

Variables	Number	Percentage (%)	*p* value
**11βHSD1 expression**			
Low	234	75.48	**< 0.001**
High	76	24.52	
**Gender**			
Male	284	91.61	0.883
Female	26	8.39	
**Age (years)**			
≤50	153	49.35	0.422
＞50	157	50.65	
**HBV**			
Negative	18	5.81	0.866
Positive	292	94.19	
**HCV**			
Negative	304	98.06	0.568
Positive	6	1.94	
**Schistosoma**			
Negative	288	92.90	0.541
Positive	22	7.10	
**Cirrhosis**			
Negative	74	23.87	0.781
Positive	236	76.13	
**Tumor size (cm)**			
≤5	119	39.27	**0.034**
＞5	184	60.73	
**Tumor number**			
Single	233	79.52	**0.048**
Multiple	60	20.48	

**Tumor encapsulation**			
None	159	53.18	**0.030**
Complete	140	46.82	
**Vascular invasion**			
Negative	205	66.99	**0.004**
Positive	101	33.01	
**PVTT**			
Negative	230	75.91	**0.044**
Positive	73	24.09	
**CTC**			
Negative	73	61.34	**0.038**
Positive	46	38.66	
**Local invasion**			
Negative	277	94.54	0.642
Positive	16	5.46	
**lymphatic invasion**			
Negative	287	98.29	0.376
Positive	5	1.71	
**Distant metastasis**			
Negative	292	97.01	0.623
Positive	9	2.99	
**Differentiation**			
Poor	111	36.27	**0.041**
Medium	148	48.37	**0.012**
Well	47	15.36	
**Endmondson-Steiner grade**			
I	74	23.87	**0.009**
II	33	10.65	**0.047**
III	108	34.84	**< 0.001**
IV	95	30.65	
**Child-Pugh stage**			

A	240	81.08	**0.047**
B	56	18.92	
**TNM stage**			
I	160	51.61	**0.046**
II	85	27.42	
III	55	17.74	
IV	10	3.23	
**BCLC stage**			
0	6	1.95	
A	130	42.21	
B	20	6.49	**0.012**
C	144	46.75	
D	8	2.60	
**Glycemia (mmol/L)**			
≤6.1	225	81.52	**< 0.001**
＞6.1	51	18.48	
**ALT (U/L)**			
≤40	192	63.79	0.506
＞40	109	36.21	
**AST (U/L)**			
≤40	167	54.93	0.087
＞40	137	45.07	
**TBIL (μmol/L)**			
≤17.1	216	74.48	0.821
＞17.1	74	25.52	
**Cholesterol (mmol/L)**			
≤5.2	208	89.66	0.439
＞5.2	24	10.34	
**ALP (U/L)**			

≤110	189	73.26	0.761
＞110	69	26.74	
**GGT (U/L)**			
≤50	104	39.39	0.308
＞50	160	60.61	
**AFP (μg/L)**			
≤20	95	32.65	< 0.001
＞20	196	67.35	
**CEA (ng/ml)**			
≤5.9	241	95.63	0.258
＞5.9	11	4.37	
**CA19-9 (U/ml)**			
≤40	233	91.73	0.987
＞40	21	8.27	
**Adjuvant TACE**			
No	284	93.42	0.106
Yes	20	6.58	

Supplementary Table 2. Univariate and Multivariate Analyses of Prognostic Factors in HCC

	Recurrence					
	Univariate analysis			Multivariate analysis		
	HR	95%CI	*p* value	HR	95%CI	*p* value
Tumor size (＞5 cm vs ≤5 cm)	0.366	0.260-0.541	< 0.001	0.453	0.303-0.677	< 0.001
Tumor number (multiple vs single)	0.459	0.335-0.630	< 0.001			
Tumor encapsulation (none vs complete)	0.375	0.276-0.509	< 0.001	0.665	0.455-0.972	0.035
Vascular invasion (positive vs negative)	0.382	0.284-0.514	< 0.001	1.210	0.601-2.436	0.039
PVTT (positive vs negative)	0.385	0.280-0.530	< 0.001	0.574	0.343-0.960	0.034
Tumor differentiation (poor vs medium-well)	2.009	1.948-2.695	< 0.001			
Endmondson-Steiner grade (I-III vs IV)	0.462	0.343-0.622	< 0.001			
Child-Pugh stage (B vs A)	0.723	0.522-1.002	0.051			
TNM stage (I vs II-IV)	0.328	0.241-0.448	< 0.001	0.342	0.173-0.676	0.002
BCLC stage (C-D vs 0-B)	0.482	0.359-0.648	< 0.001	1.696	0.876-3.282	0.017
Serum AFP (＞20 μg/L vs ≤20 μg/L)	0.556	0.398-0.778	0.001			
Glycemia (≤6.1 mmol/L vs ＞6.1 mmol/L)	0.836	0.555-1.257	0.038	0.589	0.345-1.003	0.015
11βHSD1 expression (low vs high)	2.508	1.644-3.825	< 0.001	1.538	0.927-2.557	0.007

	Survival					
	Univariate analysis			Multivariate analysis		
	HR	95%CI	*p* value	HR	95%CI	*p* value
Tumor size (＞5 cm vs ≤5 cm)	0.309	0.191-0.500	< 0.001	0.414	0.224-0.765	0.005
Tumor number (multiple vs single)	0.552	0.360-0.845	0.006	1.454	0.706-2.782	0.038
Tumor encapsulation (none vs complete)	0.294	0.193-0.448	< 0.001	0.623	0.370-1.079	0.023
Vascular invasion (positive vs negative)	0.201	0.134-0.301	< 0.001	0.350	0.106-1.157	0.015

PVTT (positive vs negative)	0.207	0.138-0.310	< 0.001	0.301	0.147-0.618	0.001
Tumor differentiation (poor vs medium-well)	2.811	1.907-4.143	< 0.001	1.455	0.902-2.347	0.024
Endmondson-Steiner grade (I-III vs IV)	0.322	0.218-0.476	< 0.001	0.624	0.391-0.995	0.048
Child-Pugh stage (B vs A)	0.703	0.450-1.097	0.121			
TNM stage (I vs II-IV)	0.229	0.147-0.358	< 0.001	0.234	0.087-0.628	0.004
BCLC stage (C-D vs 0-B)	0.339	0.227-0.506	< 0.001	2.275	0.721-7.182	0.001
Serum AFP (＞20 μg/L vs ≤20 μg/L)	0.965	0.633-1.470	0.867			
Glycemia (≤6.1 mmol/L vs ＞6.1 mmol/L)	1.244	0.703-2.201	0.045	0.423	0.206-0.868	0.019
11βHSD1 expression (low vs high)	0.136	0.055-0.334	< 0.001	2.951	1.097-7.940	0.032

Supplementary Table 3. Antibodies used in this study.

Antigens	Manufacturers	Application
11βHSD1	7754-1, Epitomics, Burlingame, CA, USA	1:1000 for WB, 1:200 for IHC
11βHSD2	Sc-20176, Santa Cruz Biotechnology, Santa Cruz, CA, USA	1:500 for WB, 1:100 for IHC
β-actin	sc-47778, Santa Cruz Biotechnology, Santa Cruz, CA, USA	1:10000 for WB
CD31	#3528, Cell Signaling Technology, Beverly, MA, USA	1:200 for IHC
CD34	#3569, Cell Signaling Technology, Beverly, MA, USA	1:200 for IHC
HIF1α	ab51608, Abcam, Cambridge, MA, USA	1:1000 for WB
VEGF	ab46154, Abcam, Cambridge, MA, USA	1:1000 for WB
Angptl4	sc-66806, Santa Cruz Biotechnology, Santa Cruz, CA, USA	1:500 for WB
MMP1	1973-1, Epitomics, Burlingame, CA, USA	1:1000 for WB
MMP2	1948-1, Epitomics, Burlingame, CA, USA	1:1000 for WB
MMP3	1908-1, Epitomics, Burlingame, CA, USA	1:1000 for WB
MMP9	1939-1, Epitomics, Burlingame, CA, USA	1:1000 for WB
MMP11	1881-1, Epitomics, Burlingame, CA, USA	1:1000 for WB
MMP12	1906-1, Epitomics, Burlingame, CA, USA	1:1000 for WB
MMP13	1923-1, Epitomics, Burlingame, CA, USA	1:1000 for WB
MMP14	2010-1, Epitomics, Burlingame, CA, USA	1:1000 for WB
MMP17	2011-1, Epitomics, Burlingame, CA, USA	1:1000 for WB
MMP21	1955-1, Epitomics, Burlingame, CA, USA	1:1000 for WB
$p21^{cip1}$	#2947, Cell Signaling Technology, Beverly, MA, USA	1:1000 for WB
$p27^{kip1}$	#3686, Cell Signaling Technology, Beverly, MA, USA	1:1000 for WB
Cyclin D1	ab134175, Abcam, Cambridge, MA, USA	1:1000 for WB
c-MYC	1472-1, Epitomics, Burlingame, CA, USA	1:2000 for WB
$p\text{-}Rb^{ser811}$	ab109399, Abcam, Cambridge, MA, USA	1:1000 for WB
Rb	sc-74562, Santa Cruz Biotechnology, Santa Cruz, CA, USA	1:500 for WB
Cyclin A2	ab32386, Abcam, Cambridge, MA, USA	1:1000 for WB
Cyclin E1	ab133266, Abcam, Cambridge, MA, USA	1:1000 for WB
$p\text{-}CDK2^{Thr160}$	#2561, Cell Signaling Technology, Beverly, MA, USA	1:1000 for WB

CDK2	#2546, Cell Signaling Technology, Beverly, MA, USA	1:1000 for WB
CDK4	#12790, Cell Signaling Technology, Beverly, MA, USA	1:1000 for WB
GLUT4	#2213, Cell Signaling Technology, Beverly, MA, USA	1:1000 for WB
HK2	#2867, Cell Signaling Technology, Beverly, MA, USA	1:1000 for WB
PKM2	#4053, Cell Signaling Technology, Beverly, MA, USA	1:1000 for WB
GAPDH	KC-5G4, KangChen Bio-tech, Shanghai, China.	1:50000 for WB
GLUT1	#12939, Cell Signaling Technology, Beverly, MA, USA	1:1000 for WB
LDHα	A1146, Abclonal, Cambridge, MA, USA	1:1000 for WB
PDH	#3205, Cell Signaling Technology, Beverly, MA, USA	1:1000 for WB
IDH1	A2169, Abclonal, Cambridge, MA, USA	1:1000 for WB
IDH2	A7190, Abclonal, Cambridge, MA, USA	1:1000 for WB
Horseradish peroxidase (HRP) conjugated anti-rabbit IgG	Jackson ImmunoResearch Laboratories, Inc. West Grove, PA, USA	1:5000 for WB
HRP conjugated anti-mouse IgG	Jackson ImmunoResearch Laboratories, Inc. West Grove, PA, USA	1:5000 for WB
Secondary antibody	Envision kit (HRP, rabbit/mouse, DAB+), DAKO	Ready-to-use for IHC
